# *Pseudomonas aeruginosa lasI*/*rhlI* quorum sensing genes promote phagocytosis and aquaporin 9 redistribution to the leading and trailing regions in macrophages

**DOI:** 10.3389/fmicb.2015.00915

**Published:** 2015-09-03

**Authors:** Angelika Holm, Thommie Karlsson, Elena Vikström

**Affiliations:** Department of Clinical and Experimental Medicine, Faculty of Medicine and Health Sciences, Linköping UniversityLinköping, Sweden

**Keywords:** host-bacteria relationship, quorum sensing, *N*-acylhomoserine lactone, innate immunity, macrophage, water homeostasis, aquaporin

## Abstract

*Pseudomonas aeruginosa* controls production of its multiple virulence factors and biofilm development via the quorum sensing (QS) system. QS signals also interact with and affect the behavior of eukaryotic cells. Host water homeostasis and aquaporins (AQP) are essential during pathological conditions since they interfere with the cell cytoskeleton and signaling, and hereby affect cell morphology and functions. We investigated the contribution of *P. aeruginosa* QS genes *lasI/rhlI* to phagocytosis, cell morphology, AQP9 expression, and distribution in human macrophages, using immunoblotting, confocal, and nanoscale imaging. Wild type *P. aeruginosa* with a functional QS system was a more attractive prey for macrophages than the *lasI/rhlI* mutant lacking the production of QS molecules, 3O-C_12_-HSL, and C_4_-HSL, and associated virulence factors. The *P. aeruginosa* infections resulted in elevated AQP9 expression and relocalization to the leading and trailing regions in macrophages, increased cell area and length; bacteria with a functional QS system *lasI/rhlI* achieved stronger responses. We present evidence for a new role of water fluxes via AQP9 during bacteria–macrophage interaction and for the QS system as an important stimulus in this process. These novel events in the interplay between *P. aeruginosa* and macrophages may influence on the outcome of infection, inflammation, and development of disease.

## Introduction

Tissue resident macrophages can be stimulated by other cells and bacterial products to move to the site of infection, engulf, and kill the bacteria. Cell migration and phagocytosis follow a distinct pattern of repetitive cycles of protrusion of wide, thin lamellipodia, and small finger-like filopodia, resulting in cell polarization with trailing and leading ends in the direction of migration. Thus, to migrate and engulf bacteria, the macrophage must rapidly change its shape and volume; this is driven by an interplay of a highly dynamic plasma membrane, reorganization of actin cytoskeleton and a variety of signaling molecules including phospholipase C, phosphoinositide 3-kinase, Rho family of small GTPases, Ca^2+^ mobilization (Nourshargh and Alon, 2014), fluxes of monovalent ions (Na^+^, K^+^, Cl^−^), and not the least transport of water across the membrane. Although water molecules can diffuse sparsely across the lipid bilayer, membrane water channel proteins, aquaporins (AQP) are key players in this process (Verkman, 2005; Loitto et al., 2009).

Today, 13 mammalian water channels AQP0-AQP12 are known and divided in three different subgroups based on their substrate permeability: orthodox AQP exclusively permeable to water; aquaglyceroporins transporting water, glycerol, and some other small neutral solutes; and subcellular AQP (S-aquaporins, or superaquaporins) (Benga, 2012). A common feature of the AQP is that its water-transporting ability can be blocked by mercury (Hg^2+^) through its binding to a cysteine thiol localized in the pore (Savage and Stroud, 2007). In human leukocytes, the main aquaglyceroporin AQP9 (Ishibashi et al., 2009) localizes at the leading edge in migrating cells and is required for proper migration (Loitto et al., 2002, 2009). Water fluxes through AQP regulate cell shape and formation of membrane protrusion formation; the mechanism behind this relies on localized influx of water that pushes the membrane outwards allowing the actin cytoskeleton to remodel and elongate and thereby change the cell shape (Karlsson et al., 2013a,b). The involvement of AQP9 has also been implicated in LPS-induced water accumulation in brain and blood-brain barrier perturbation (Wang et al., 2009). In addition, AQP has been identified as one of several universal markers of chronic inflammation in patients with autoimmune diseases (Mesko et al., 2010). Thus, there is a multiple evidence for the importance of water homeostasis and AQPs during infection, inflammation, and bacteria–host cell communication, but the details of the cellular events remain to be further elucidated.

During phagocytosis, macrophages, and neutrophils must rapidly change shape and directional movement, and through their highly flexible membrane and dynamic cytoskeleton be able to form protrusions. Macrophages become activated by many bacterial products, such as formylated peptides and LPS, and more recently bacterial quorum sensing (QS) molecules have been recognized as a novel category of chemoattractants and modulators of phagocytic activity in leukocytes. Thus, it was shown that human neutrophils were stimulated by and migrated toward *N*-acylhomoserine lactone (AHL) molecules (Zimmermann et al., 2006; Karlsson et al., 2012). Moreover, the long-chain fatty acid AHL, 3O-C_12_-HSL stimulate the uptake of *Escherichia coli* by neutrophils (Wagner et al., 2007) and yeast particles by macrophages (Vikström et al., 2005).

The QS signaling system primarily enables bacteria to pass on information about population density and collectively regulate the expression of an ensemble of genes (Williams and Cámara, 2009; Rutherford and Bassler, 2012). The Gram-negative pathogen *P. aeruginosa* harbors at least three subordinated QS systems: two of LuxI/LuxR-type which are *N*-acylhomoserine lactone (AHL) dependent and one quinolone-type system (PQS). In the first, the LuxI homolog LasI synthesizes the diffusible *N*-3-oxo-dodecanoyl-*L*-homoserine lactone (3O-C_12_-HSL) that is recognized by the LuxR homolog, cytoplasmic receptor LasR (Moré et al., 1996; Parsek et al., 1999). In the second, the LuxI homolog RhlI produces another AHL, *N*-butyryl-*L*-homoserine lactone (C_4_-HSL) that is detected by the cytoplasmic receptor RhlR (Ochsner et al., 1994; Pearson et al., 1995). LasR and RhlR regulate transcription and maintain the activity of about 300 genes in the large *P. aeruginosa* genome. These multiple genes encode the production of rhamnolipids, surface-active molecules important for the late stage of biofilm formation (Ochsner et al., 1994; de Kievit, 2009), and extracellular virulence factors (elastases, proteases, lectins, pyocyanin, and exotoxin A) that can destroy host tissues and initiate infection and inflammation (Gambello and Iglewski, 1991; Toder et al., 1991; Gambello et al., 1993; Schuster et al., 2003). Bacterial QS can also elicit inter-kingdom signaling by affecting the behavior of eukaryotic cells, which has been addressed by many researchers (Jarosz et al., 2011; Teplitski et al., 2011; Holm and Vikström, 2014).

The aim of this study was to investigate the contribution of *P. aeruginosa* QS genes *lasI/rhlI* to phagocytosis, cell morphology, AQP9 expression, and distribution in macrophages, using immunoblotting, confocal, and nanoscale imaging.

## Materials and methods

### Ethics statement

The study was carried out according to the Declaration of Helsinki. Human blood and buffy coat was collected by employees at the Blood Bank, Linköping University Hospital, Sweden. A written agreement for research use of blood was obtained from all donors. The study did not require ethical approval according to paragraph 4 of the Swedish law (2003:460; http://www.lagboken.se/dokument/Lagar-och-forordningar/4060/Lag-2003_460-om-etikprovning-av-forskning-som-avser-manniskor?id=64991) on Ethical Conduct in Human Research, since blood donation is assumed as negligible risk to the donors and only anonymized blood samples were delivered to the researchers.

### Human primary monocyte-derived macrophages

Monocytes were obtained from buffy coat from healthy blood donors (Blood Bank, Linköping University Hospital). The buffy coat was mixed 50/50 with cold 0.9% NaCl and a leukocyte concentrate was obtained using a Lymphoprep gradient (Axis Shield, Oslo, Norway) by centrifugation for 40 min at 480 × g at room temperature (RT). The mononuclear cell layer was collected, washed thrice in cold PBS, pH7.3 with 0.1 IE/ml heparin (LEO Pharma, Ballerup, Denmark) and thrice in cold calcium-free Krebs-Ringer Glucose buffer, KRG (120 mM NaCl, 4.9 mM KCl, 1.2 mM MgSO_4_, 8.3 mM KH_2_PO_4_, 10 mM glucose) to remove the density gradient. Cells were seeded in Dulbecco's modified Eagle's medium, DMEM, with 25 mM HEPES, 100 U/ml penicillin and 100 μg/ml streptomycin (Life Technologies, Grand Island, NY) and left to adhere for 1.5–2 h at 37°C in 5% CO_2_. Unbound cells were washed out with calcium-containing KRG, KRG+Ca^2+^ (as above with addition of 1 mM CaCl_2_) at 37°C. The adherent monocytes were allowed to differentiate in DMEM with 10% human serum (pooled from five healthy donors, Blood Bank, Linköping University Hospital) and 80 μM L-glutamine (Life Technologies). After 7–8 days, the cells were considered to be differentiated macrophages, based on their morphology and phagocytic activity. For experiments, macrophages were detached with trypsin-EDTA (Life Technologies), washed, counted, and reseeded at concentration 10^6^ cells/well in six-well plates for further immunoblotting or at 5 × 10^4^ on glass coverslips (thickness 0.17 ± 0.01, 13 mm-diameter; Karl Hecht Assistent, Sondheim, Germany) for phagocytosis and imaging. Cells were left to adhere in DMEM as above for 2 h at 37°C in 5% CO_2_, and then starved overnight in DMEM with 25 mM HEPES without serum and antibiotics.

### Bacterial strains

Two *P. aeruginosa* strains used were wild-type PAO1 and its double mutant PAO1 *lasI-/rhlI-* which is also called PAO1-JP2, PAO Δ*rhlI*::Tn501 *lasI*::Tc^*r*^, Hg^*r*^ (Brint and Ohman, 1995; Pearson et al., 1997) and defective in the synthesis of QS molecules 3O-C_12_-HSL and C_4_-HSL. Both strains were constitutively expressing the stable green fluorescent protein, GFP, and were a kind gift from Prof. Thomas Bjarnsholt (University of Copenhagen, Denmark). Bacteria were grown in Luria-Bertani (LB) liquid medium or on agar plates containing 150–200 μg/ml carbenicillin to maintain a stable expression of GFP; tetracycline (50 or 300 μg/ml) and mercuric chloride (7.5 or 15 μg/ml) were added (all from Sigma Aldrich, St. Louis, MO) to maintain the *lasI-/rhlI-* mutations.

### AHL synthesis

Two AHL, long-chain fatty acid 3O-C_12_-HSL (MW297), and short-chain C_4_-HSL (MW171) were synthesized by Prof. Peter Konradsson and co-workers (Linköping University, Sweden) as previously described (Chhabra et al., 2003). Both AHL are structurally and functionally identical to those obtained from *P. aeruginosa* cultures; they were checked for identity and purity by HPLC, and their biological activity as QS molecules was validated by the bioassay described earlier (Surette and Bassler, 1998). Stock AHL were dissolved in dimethylsulfoxide (DMSO).

### Bacterial infection and treatment with AHL and AQP blockers

Before infection, bacteria were grown in LB medium overnight at 37°C with shaking, re-suspended in 0.9% NaCl to an optical density of 0.09 at 600 nm, OD_600_ (1 × 10^8^ CFU/ml) and further diluted in DMEM with 25 mM HEPES without serum and antibiotics. Macrophages, at a concentration 10^6^ cells/well or 5 × 10^4^ on glass coverslips, were infected with bacteria at multiplicities of infection (MOI) 1, 10, and 100 in fresh DMEM, incubated for 1 h at 37°C in 5% CO_2_ and further proceeded for the phagocytosis assay, imaging or immunoblotting. To examine the functional complementation of phagocytosis, macrophages were pretreated with a mixture of 25 μM C_4_-HSL and 50 μM 3O-C_12_-HSL (in 0.02% DMSO) for 4 h before infection at MOI 10. To assess the role of AQP, two kinds of water influx inhibitors were used: HgCl_2_, which blocks water transport activity of most AQP through thiol binding to a pore-localized cysteine (Savage and Stroud, 2007); and HTS13286 a specific peptide inhibitor of AQP9 (Jelen et al., 2011; Karlsson et al., 2013a; Wacker et al., 2013). Macrophages were pretreated with 1 or 5 μM HgCl_2_ (Sigma Aldrich) or 25 μM HTS13286 (Maybridge, Cornwall, UK) for 20 min. As vehicle for HTS13286, 0.25% DMSO was used.

### Phagocytosis assay

**Figure 1:**
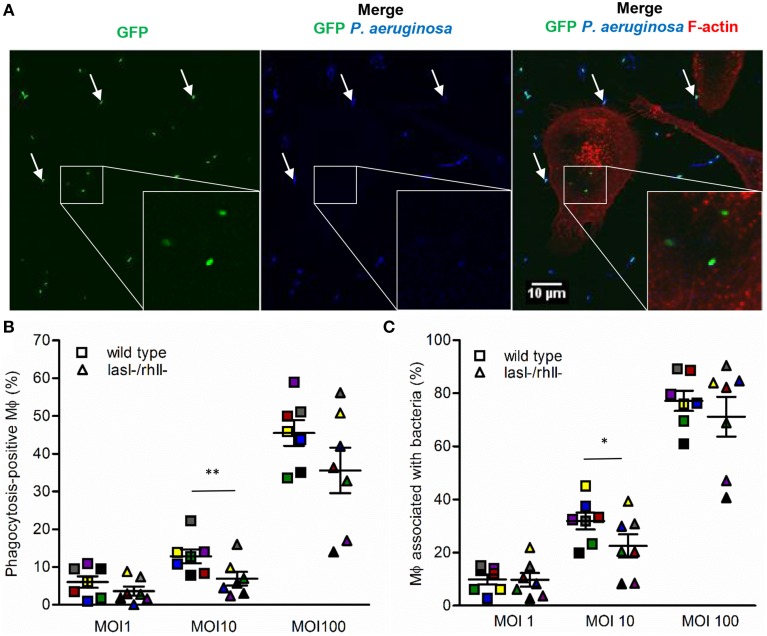
**Binding and phagocytosis of wild type ***P. aeruginosa*** and its ***lasI-/rhlI-*** mutant**. **(A)** Macrophages were infected with GFP (green) wild type bacteria or *lasI-/rhlI-* mutant, stained for *P. aeruginosa* (blue) and F-actin (red), and analyzed by LSCM. White squares show three ingested bacteria distinguished by sole GFP (green). White arrows point to bound bacteria recognized by combined GFP (green) and *P. aeruginosa* (blue). Bar 10 μm. **(B)** Quantification of phagocytosis presented as the percentage of phagocytic-positive (containing ingested bacteria) cells among total macrophages. **(C)** Quantification of binding presented as the percentage of cells containing bound and ingested bacteria (associated bacteria) among total macrophages. Shown are the mean ± SE of seven independent experiments performed at separate days from different donors (color coded). The means ± SE are based on 100–200 cells for each condition per experiment. Significant differences were considered at ^*^P < 0.05 and ^**^P < 0.01, as calculated by two-tailed paired Student's *t*-test.

Samples on glass cover slips were washed with KRG+Ca^2+^, fixed with 4% paraformaldehyde (Sigma Aldrich) for 20 min at RT and blocked in 1% BSA. To label non-ingested and surface-attached bacteria, the samples were incubated for 1 h at RT with rabbit anti-*Pseudomonas* antibodies (#PAI-73116, Thermo Scientific, Rockford, IL), diluted 1:800, washed and incubated with Alexa Fluor 635-conjugated goat anti-rabbit antibodies (Life Technologies), 1:1000 for 1 h at RT. The cells were permeabilized in 0.1% Triton X-100 in PBS (Sigma Aldrich) for 5 min at RT, washed again and stained for 20 min at RT with Alexa Fluor 568-conjugated phalloidin for cytoskeleton F-actin labeling (Life Technologies), diluted 1:40 in PBS from 200 units/ml methanol stock solution. After washing, the speciments were mounted in ProLong Gold antifade reagent (Life Technologies) and examined through a 63 × oil immersion objectives with NA 1.40 in a fluorescence microscope Zeiss Axio Observer Z1 with confocal system Zeiss LSM700 and Zeiss ZEN software (Carl Zeiss, Jena, Germany). Random images were chosen through the red channel (F-actin) to avoid biases for only selecting phagocytic cells with bacteria. Completely ingested bacteria were distinguished by their sole GFP fluorescence (green); bound and non-ingested ones were recognized by combined GFP (green) and Alexa Fluor 647 (blue) color (Figure 1A). Macrophages containing ingested bacteria were counted as phagocytosis-positive cells (Figure 1A, shown in white squares and inserts); and macrophages with adherent (Figure 1A, pointed by white arrows) and/or ingested bacteria were counted as macrophages associated with bacteria. To quantitate, Fidji software (NIH, Bethesda, MD) was used; binding- and phagocytic activities were presented as the percentage of phagocytosis-positive macrophages among total cells. The results from four-seven independent experiments, performed on separate days and from different blood donors were expressed as the mean ± SE; about 100–200 macrophages per sample were counted. The images and measurements were taken by two independent observers or by blinding of the samples.

### LSCM, SIM, and STED imaging

The specimens for imaging were fixed and stained to label non-ingested or surface-attached bacteria, as described above for the phagocytosis assay. Then, the samples were stained with rabbit anti-AQP9 antibodies (#ab84828-100, Abcam, UK) and Atto647N (fluorophore for STED) secondary antibodies (Active Motif, Carlsbad, CA), and mounted in ProLong Gold antifade reagent (Life Technologies). For laser scanning confocal microscopy (LSCM), the specimens were examined through a 63 × oil immersion objectives with NA 1.40 in a fluorescence microscope Zeiss Axio Observer Z1 with confocal system Zeiss LSM 700 and Zeiss ZEN software (Carl Zeiss, Jena, Germany). For structured illumination microscopy (SIM), yielding resolution of around 100 nm which is double, than could be achieved in LSCM, the samples were examined in a fluorescence microscope Zeiss LSM 780 with ELYRA S.1 system (Carl Zeiss, Jena). For nanoscale visualization, allowing capture very fine structural details with a resolution of around 20–40 nm, which is 5–10 times higher than can be achieved in LSCM, the specimens were examined in a Leica TCS STED Stimulated Emission Depletion confocal microscope with a pulsed IR-laser and a 100 × oil immersion objective (Leica Microsystems, Mannheim, Germany). These advanced techniques are based on either probing the samples with spatially modulated illumination or switching fluorophores on and off sequentially in time (Blom and Brismar, 2014). Fluorescence intensity, cell area, and length were measured and quantified using the Fidji software (NIH). The images and measurements were taken by two independent observers or by blinding of the samples.

### Preparation of total cell lysates, SDS-PAGE, and immunoblotting

Samples were rinsed with PBS, pH 7.6 and lysed with cold RIPA buffer (1% NP-40, 1% deoxycholic acid sodium salt, 0.1% SDS, 150 mM NaCl, 10 mM Tris pH 7.4, 10 mM EDTA pH 8.0 dissolved in PBS) containing 25 U nuclease (Thermo Scientific), 1 mM phenyl-methyl-sulfonyl-fluoride, 1 mM Na_3_VaO_4_, 25 mM NaF (Sigma), protein inhibitors Complete (Roche Diagnostics, Mannheim, Germany). Cell lysates were homogenized through a 21-gauge needle, centrifuged at 18,000 g for 30 min at 4°C, and the supernatants collected. The protein concentrations in lysates were measured with the Bio-Rad D_C_ protein assay (Bio-Rad Laboratories, Hercules, CA). The samples were diluted in Laemmli sample buffer at equal protein concentrations, heated for 5 min at 95°C and loaded on 4–12% SDS-polyacrylamide gels (Lonza, Rockland, ME). After separation, proteins were electrophoretically transferred to a PVDF membrane (Millipore, Bedford, MA); the quality of the transfer was monitored by Ponceau S staining (Sigma Aldrich). The membranes were blocked in 5% non-fat milk in PBS pH 7.6, with 0.18% Tween-20 for 1 h at RT and incubated overnight at 4°C with rabbit anti-AQP9 antibodies (#ab85910 Abcam), diluted 1:2000 or mouse anti-GAPDH antibodies (Millipore, Temecula, CA) in blocking buffer. After washing, they were incubated for 1 h at RT with IRDye 800CW goat anti-rabbit or IRDye 680CW goat anti-mouse antibodies (LI-COR Biosciences, Cambridge, UK), diluted 1:10,000 and washed extensively. The signals were detected and quantified by Odyssey CLx and the Image Studio software (LI-COR).

### mRNA array analysis

Cells were rinsed with PBS, pH 7.6 and lysed with TRIzol (Life Technologies) to obtain total RNA. The Affymetrix human arrays and mRNA expression profiling were performed by Atlas Biolabs GmbH (Berlin, Germany).

### Statistical analysis

Data are presented as mean ± SE. Data from the phagocytosis assays were analyzed using paired, two-tailed *t*-test. Whole cell and over the cell AQP9 fluorescence intensity were analyzed using unpaired, two-tailed *t*-test when comparing control vs. infected samples and one-tailed *t*-test for comparison of wild type and *lasI-/rhlI-* mutant. The numbers (*n*) are specified in the figure legends. *P* < 0.05 were considered significant.

## Results

### More effective binding and phagocytosis of wild type *P. aeruginosa* than of its *lasI-/rhlI-* mutant

QS signal molecules with a long fatty-acid chain and an intact ring, e.g., 3O-C_12_-HSL and 3O-C_10_-HSL, can by themselves affect leukocytes chemotaxis (Zimmermann et al., 2006; Karlsson et al., 2012) and phagocytosis of yeast and *E.coli* (Vikström et al., 2005; Wagner et al., 2007). In the present phagocytosis model using *P. aeruginosa* bacteria as preys, we studied comparatively how wild-type and *lasI-/rhlI-* mutant, defective in the synthesis of 3O-C_12_-HSL and C_4_-HSL are phagocytized by human macrophages (Figure 1A). After 1-h infection, we observed an increased binding and phagocytic activity in macrophages treated with wild-type bacteria compared to with *lasI-/rhlI-* mutant at all MOI (Figures 1B,C). Quantitatively, wild-type bacteria at MOI 10 induced a significant, 1.42- and 1.86-fold greater binding and phagocytic capacity, respectively, in contrast to the QS defective mutant. At MOI 1 and 100, the patterns of macrophage activity to adhere and engulf wild-type *P. aeruginosa* were also increased, but not significantly compared to mutant (Figures 1B,C). This can be explained by a MOI-dependent effect on the capacity and effectiveness of migration, binding, and phagocytosis, which rely on an optimal concentration of the prey, the chemical nature, and gradient of chemotactic ligands released by bacteria, resulting in either attraction or repulsion of macrophages. The number of adherent and ingested bacteria per individual phagocytizing macrophage were very similar (Figures S1A,B).

**Figure 2:**
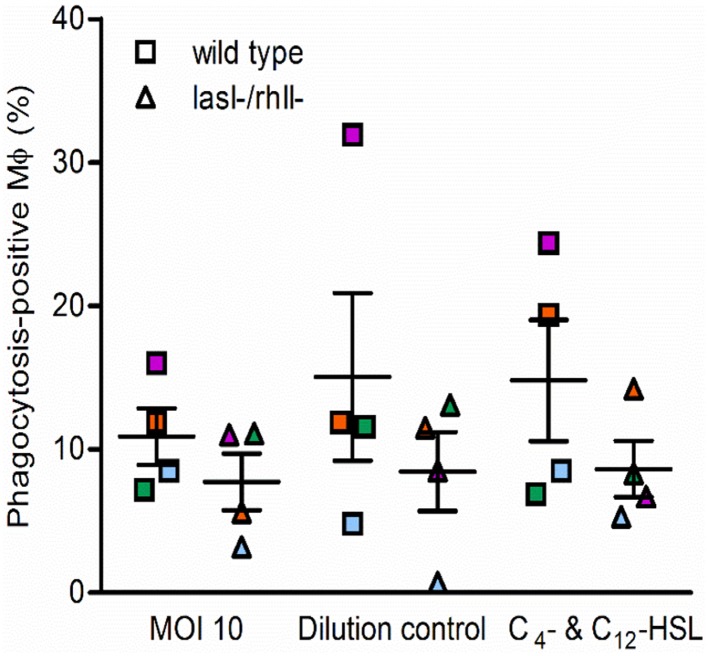
**Functional complementation of phagocytosis with AHL**. Macrophages were pretreated with 25 μM C_4_-HSL and 50 μM 3O-C_12_-HSL (C_4_- C_12_-HSL), or 0.02% DMSO as a vehicle control (Dilution control), or not-pretreated (MOI 10) for 4 h before 1-h infection with *P. aeruginosa* wild type or *lasI-/rhlI-* mutant, at MOI 10. Quantification of phagocytosis presented as the percentage of phagocytic-positive (containing ingested bacteria) cells among total macrophages. Shown are mean ± SE of four independent experiments performed at separate days from four different donors (color coded). The means ± SE are based on 100–200 cells for each condition per experiment.

We next determined whether phagocytosis activity could be functionally complemented by pre-treatment of macrophages with synthetic C_4_-HSL and 3O-C_12_-HSL before infection with either wild type or *lasI-/rhlI-* mutant. As shown in Figure 2, in one of four independent experiments, marked with orange, stimulation with AHL was able to elevate phagocytosis and rescue the difference seen in the phagocytic activity between the wild type and the mutant. This partial inconsistence can be explained by donor-specific variations in the macrophages that can display different capacities and functions, including migration and phagocytosis. Taken together, this suggests that wild type *P. aeruginosa* is more attractive prey for macrophages or stimulate their own ingestion compared to the strain with a mutation in the *lasI* and *rhlI* genes and lacking synthesis of 3O-C_12_-HSL and C_4_-HSL and hence many virulence factors.

### AQP9 protein levels are increased in macrophages infected with *P. aeruginosa*

**Figure 3:**
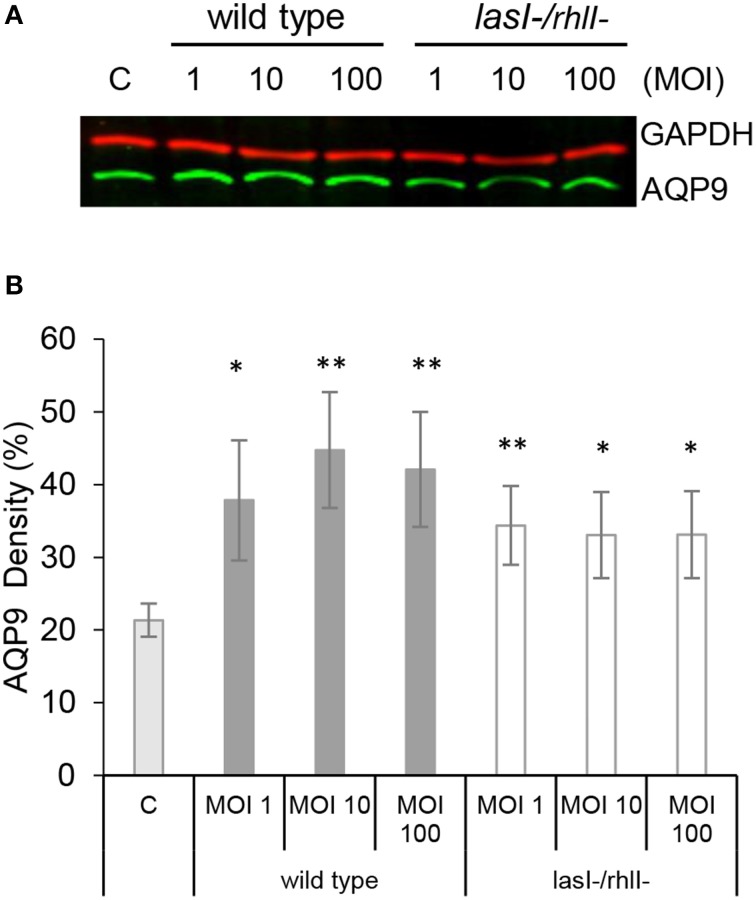
**AQP9 protein levels are increased in macrophages infected with ***P. aeruginosa*****. **(A)** Macrophages were either non-infected (C) or infected with wild type *P. aeruginosa* or *lasI-/rhlI-* mutant at MOI 1, 10, and 100 for 1 h. Total cellular protein extracts were analyzed with Western blot for AQP9, 31 kDa (lower, green bands) and GAPDH, 36 kDa as a loading control (upper, red bands). The blots are from one representative of four independent experiments. **(B)** Densitometric analysis. Values are mean ± SE percentage of AQP9 density relative to the loading GAPDH control from four independent experiment performed at separate days from four different donors. Significant differences are indicated when ^*^P < 0.05 and ^**^P < 0.01, as analyzed by Student's *t*-test.

For migration into tissues, human leukocytes must to be able to quickly change their morphology and volume, which is largely driven by water fluxes across the membrane through aquaporins, primarily AQP9 in human leukocytes (Loitto et al., 2002, 2009; Karlsson et al., 2013a). Since macrophage phagocytosis of bacteria with intact QS system was increased, we addressed whether AQP9 was involved in this process by investigating the effects of the wild type *P. aeruginosa* and *lasI-/rhlI-* mutant on the protein expression levels of AQP9 in macrophages. This was analyzed by immunoblotting for AQP9 and GAPDH, as a loading control (Figure 3A), with subsequent quantification of the density of the specific bands (Figure 3B). Here, treatment with wild type *P. aeruginosa* and *lasI-/rhlI-* mutant at all MOI caused a significantly increased expression of AQP9 compared to non-infected control macrophages. Wild type bacteria seems to cause a more pronounced increase in the expression level of AQP9 than its QS defective mutant, but no significant differences were found. To assess the relationship between protein and mRNA expression levels, we assessed mRNA for all AQP types, AQP0-12. After infection with wild type *P. aeruginosa* and *lasI-/rhlI-* mutant for 1 h, mRNA levels were slightly affected for AQP0, 1, 2, 3, 8, 11, and 12, with exception for AQP4, 5, 6, 7, 9, and 10 that maintained near to the basal expression level (Table S1).

### Effects of *P. aeruginosa* on the cellular distribution of AQP9

**Figure 4:**
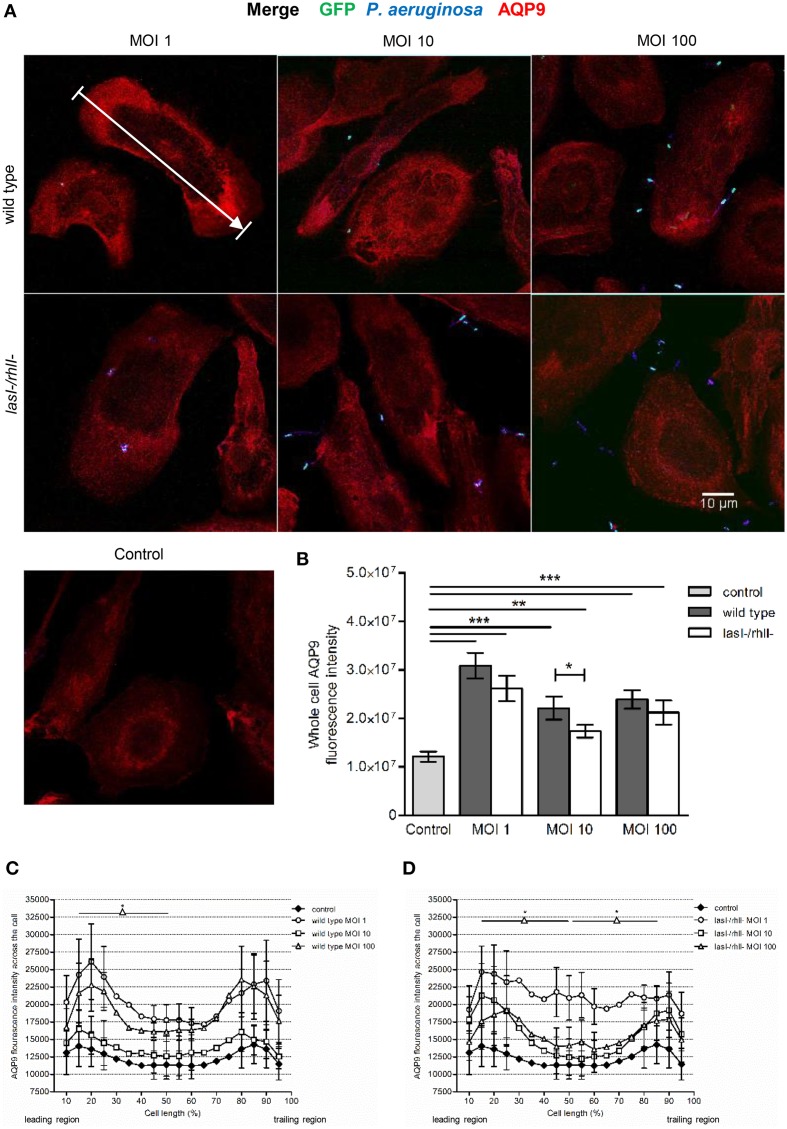
**Effects of ***P. aeruginosa*** on the cellular distribution of AQP9**. **(A)** Macrophages were infected with GFP (green) wild type *P. aeruginosa*, the *lasI-/rhlI-* mutant, or non-infected (Control), stained for *P. aeruginosa* (blue), AQP9 (red) and analyzed by LSCM. The data are from one of at least three independent experiments. Bar 10 μm. **(B)** Quantification of AQP9 immunofluorescence intensity measured as a total integrated intensity of whole macrophage area, of at least 30 cells for each condition. Data are from at least three experiments performed at separate days from different donors. Columns represent means ± SE. Significant differences are indicated when ^**^P < 0.01 and ^***^P < 0.0001, as analyzed by two-tailed unpaired Student's *t*-test. **(C,D)** Quantification of AQP9 immunofluorescence intensity profile measured across the cells in the direction of polarization as indicated by white arrow in **(A)**. Significant differences are indicated when ^*^P < 0.05, as analyzed by one-tailed unpaired Student's *t*-test.

During locomotion, the cellular distribution and dynamics of AQP9 has been shown to be crucial in promoting protrusion activity by accumulation in membrane domains and preceding formation of filopodia, blebs, and lamellipodia (Loitto et al., 2002; Karlsson et al., 2011, 2013a,b). Therefore, we investigated whether *P. aeruginosa* affected the cellular distribution and localization of AQP9 using confocal imaging (Figure 4A). The control, non-infected macrophages displayed low levels of AQP9 in the cytoplasmic region. But after 1-h infection with wild type *P. aeruginosa* and *lasI-/rhlI-* mutant at all MOI, we observed remarkable alterations in intensity and localization of AQP9. Here, the whole cell AQP9 fluorescence intensity was significantly higher in all infected macrophages compared to untreated control (Figure 4B). Interestingly, wild type *P. aeruginosa* at MOI 10 lead to significantly increased whole cell AQP9 fluorescence intensity than the *lasI-/rhlI-* mutant. In general, bacteria-infected macrophages became more polarized in their morphology, and measurements of AQP9 fluorescence intensity over the macrophages in the direction of polarization (as indicated by white arrow in Figure 4A), demonstrated significant re-localization of AQP9 in infected cells compared to uninfected controls. The infected cells displayed a more AQP9 in the front and the back of the cell, whereas the control have a smoother, more evenly distributed profile of AQP9 (Figures 4C,D). Taken together, *P. aeruginosa* infection lead to increased cellular distribution and re-localization of AQP9 to the leading and trailing regions in human macrophages, and bacteria with functional QS system may contribute more to this process.

### *P. aeruginosa* affects cell morphology in macrophages

**Figure 5:**
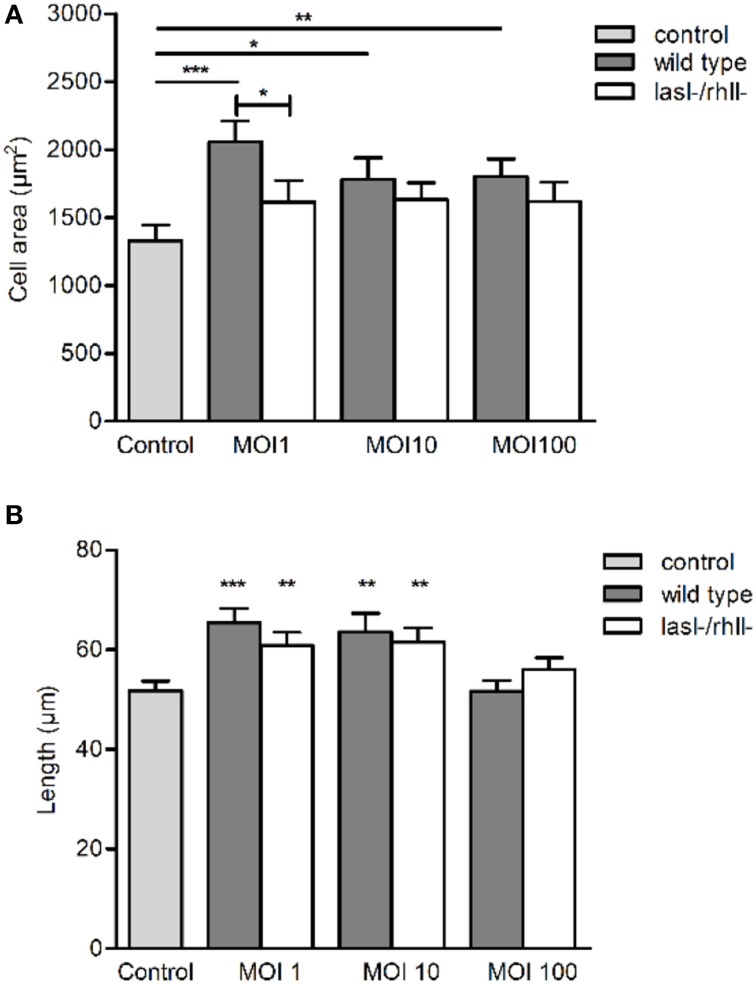
*****P. aeruginosa*** affects cell morphology in macrophages**. Macrophages were infected with wild type *P. aeruginosa*, the *lasI-/rhlI-* mutant or were non-infected (Control), stained, and imaged as in Figure 4A. **(A)** Quantification of the macrophage cell area. **(B)** Quantification of approximated macrophage length (as indicated by white arrow in Figure 4A in the direction of polarization). Columns represent means ± SE of three different experiments with three donors. Significant differences are indicated when ^*^P < 0.05, ^**^P < 0.01 and ^***^P < 0.0001, as analyzed by two-tailed unpaired Student's *t*-test.

Since *P. aeruginosa* infection caused increased cellular distribution and re-localization of AQP9 in macrophages, parallel changes in their morphology and volume driven by water fluxes across the AQP may occur. The cell area was significantly larger at all MOIs after infection with wild type bacteria compared to uninfected control (Figure 5A). *P. aeruginosa lasI-/rhlI-* mutant had smaller effect on the cell area and no significant differences compared to uninfected control (Figure 5A). The approximated cell length, measured in the direction of polarization, shown in Figure 4A, was significantly greater in macrophages infected with wild type *P. aeruginosa* and *lasI-/rhlI-* mutant at MOI 1 and 10 compared to untreated control macrophages (Figure 5B). These data confirm that the *P. aeruginosa*-induced increase in cellular distribution and re-localization of AQP9 in macrophages are paralleled with changes in cell size.

### Nanoscale visualization of AQP9 in macrophages during *P. aeruginosa* infections

**Figure 6:**
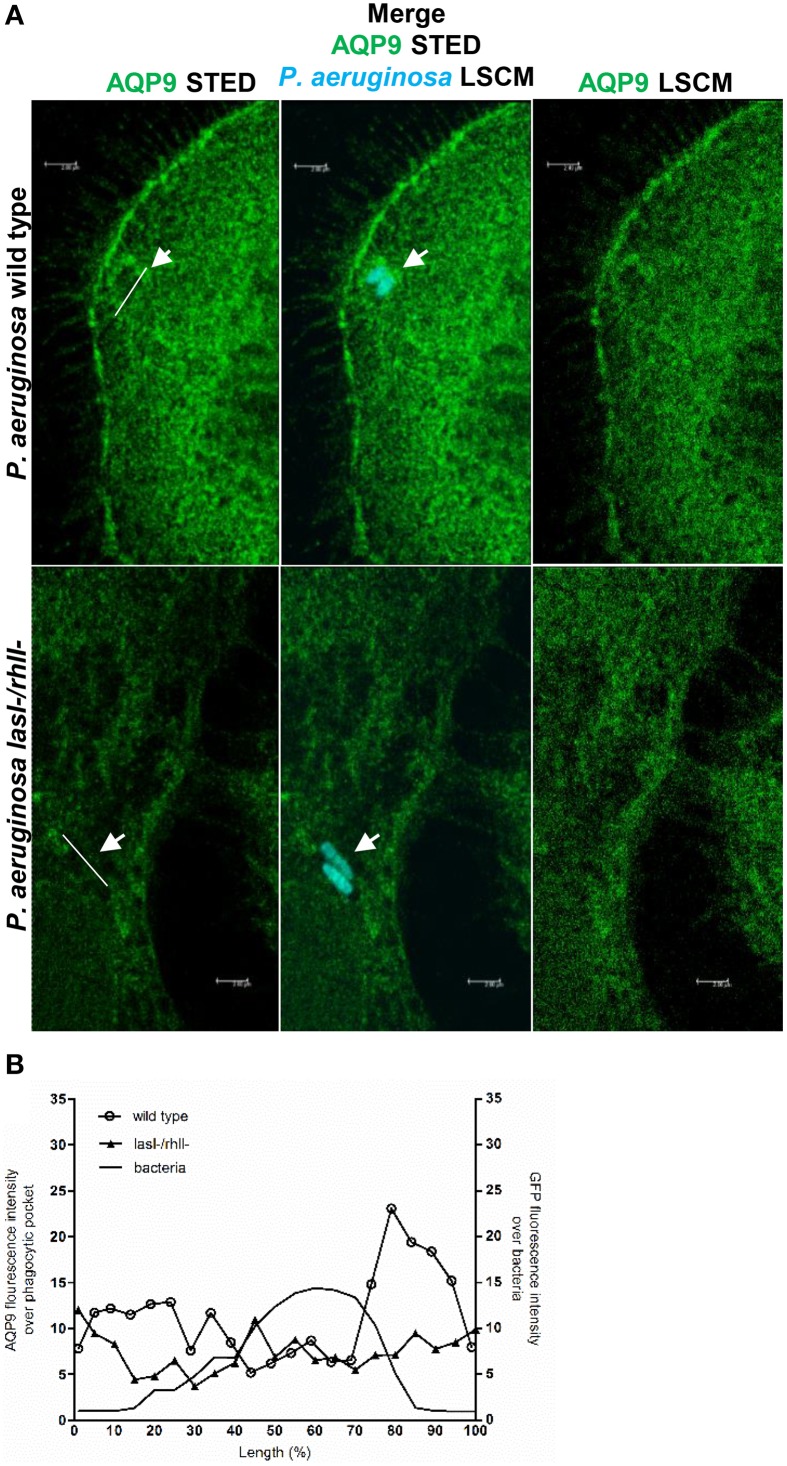
**Nanoscale visualization of AQP9 in macrophages during ***P***. ***aeruginosa*** infections**. **(A)** Macrophages were infected with GFP-labeled (cyan) wild type *P. aeruginosa* or the *lasI-/rhlI-* mutant or non-infected (not shown). Cells were stained for non-ingested *P. aeruginosa* (not shown), and AQP9 (green), and analyzed by STED. Bacteria shown at these images were completely ingested (cyan). White arrows points to pocket-like AQP9 accumulation around completely ingested wild type bacteria. Bar 2 μm. **(B)** Mean AQP9 intensity profiles over phagocytic pockets and mean GFP intensity profile over bacteria from representative images **(A)** measured as indicated by the white lines.

To further study the details of *P. aeruginosa*-induced alterations in AQP9 distribution and localization in human macrophages, we used two novel super-resolution imaging techniques, giving much sharper images. These were structured illumination microscopy (SIM), yielding resolution of around 100 nm (Figure S2), and stimulated emission depletion (STED), allowing a further increased resolution of around 20–40 nm (Figure 6A). After 1-h infection with wild type *P. aeruginosa* at MOI 10, we observed more distinct AQP9 localization in the cytoplasm at peripheral regions in vicinity of leading- and trailing- edges and in protruding structures, *i.e.*, in filopodia and lamellae. In addition, the wild type strain triggered a pocket-like AQP9 accumulation around intracellular bacteria. By contrast, the *lasI-/rhlI-* mutant induced smaller and modest effects on the AQP9 nanostructure (white arrow Figures 6A,B). Thus, super-resolution imaging revealed distinct details in nanoscale structural organization of AQP9 in macrophages during phagocytosis of wild type *P. aeruginosa* and the *lasI-/rhlI-* mutant.

### Effect of water fluxes inhibitors on phagocytosis of *P. aeruginosa* by macrophages

**Figure 7:**
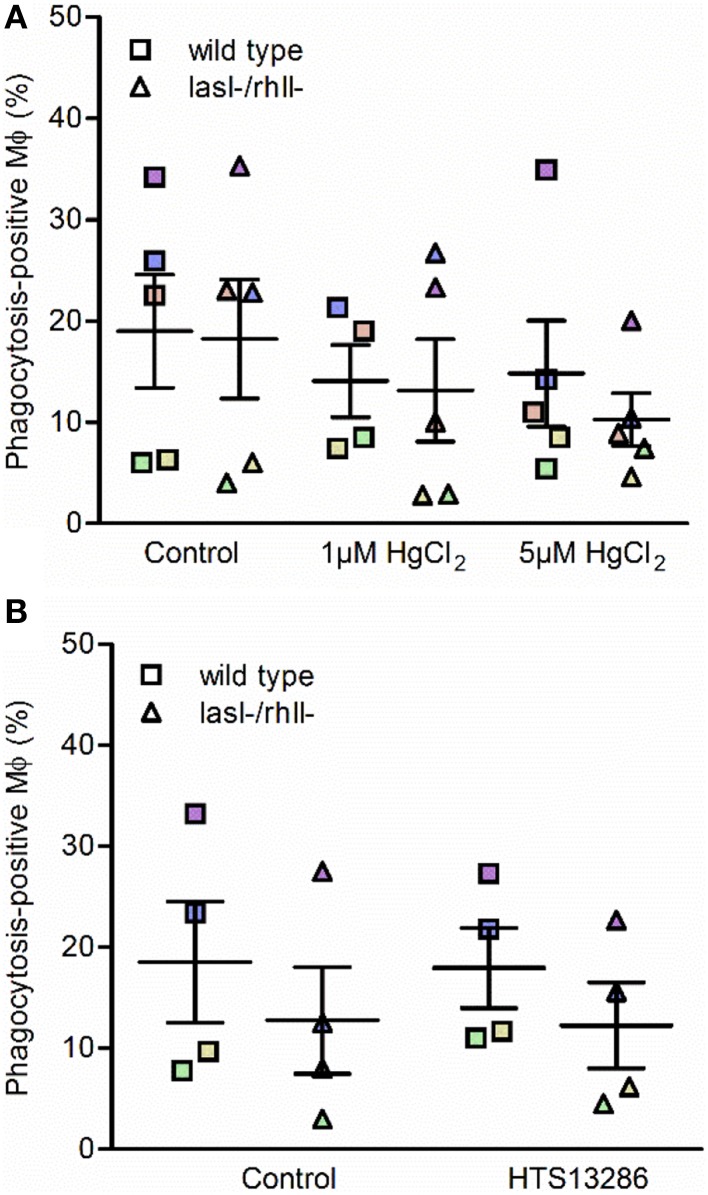
**Effect of water fluxes inhibitors on phagocytosis of ***P***. ***aeruginosa*** by macrophages**. Macrophages were pretreated with **(A)** 1 or 5 μM HgCl_2_, or PBS as a control; **(B)** 25 μM HTS13286 or 0.25% DMSO as vehicle control for 20 min. Wild type *P. aeruginosa* or the *lasI-/rhlI-* mutant at MOI 10 were added for 1 h of infection and phagocytic activity of macrophages were evaluated. Shown are the mean ± SE of the percentage of phagocytosis-positive macrophages. Data are from individual independent experiments performed at separate days from 4 to 5 different donors (color coded).

Next, we investigated whether inhibition of water transport via AQP and particularly AQP9 influenced the phagocytosis of *P. aeruginosa* by macrophages. To do so, macrophages were pretreated for 20 min with 1 or 5 μM HgCl_2_ to block most AQP and 25 μM HTS13286 to inhibit AQP9, and PBS or 0.25% DMSO as vehicle control. We observed that the inhibition of water fluxes via most AQP only slightly prevented the phagocytosis of bacteria (Figure 7A), while AQP9 blocker failed to get a clear effect (Figure 7B). Looking at the responses of the cells from individual donors, those marked with violet, blue, and brown, there was an inhibitory effect at 5 μM HgCl_2_. For the specific AQP9 inhibitor, this was seen only for the “high responder” violet cells. These data suggest that water transport across the membrane through AQP only partly facilitated the phagocytosis of *P. aeruginosa* by macrophages, suggesting that other crucial cellular signaling pathways were engaged in the event.

## Discussion

**Figure 8:**
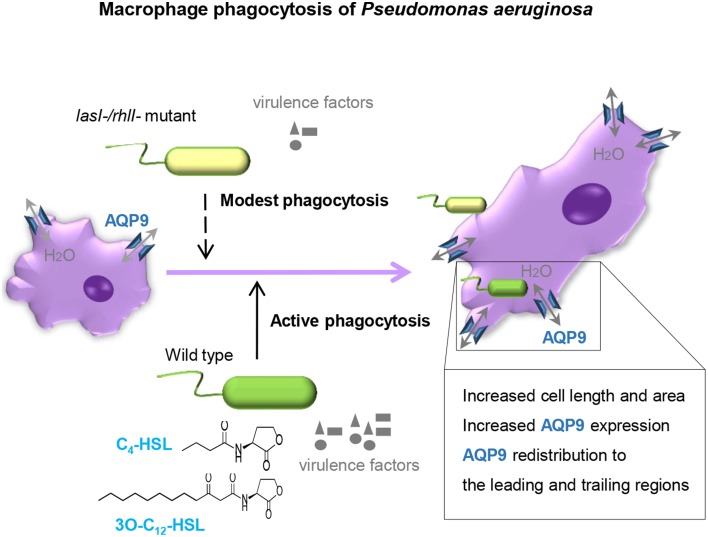
**Proposed model**. *P. aeruginosa lasI*/*rhlI* quorum sensing genes promote phagocytosis and AQP9 redistribution to the leading and trailing regions in macrophages. The phagocytosis of *P. aeruginosa* by macrophages seems more effective when QS genes *lasI* and *rhlI* responsible for synthesis of two QS molequles 3O-C_12_-HSL and C_4_-HSL and associated virulence factors are fully functional. Moreover, the *P. aeruginosa* infections results in elevated AQP9 expression and relocalization to the leading and trailing regions in macrophages, increased cell area, and length; here, bacteria with a functional QS system *lasI/rhlI* achieved stronger responses.

The outcome of infections and development of disease depend on both host defense and bacterial traits, and where QS plays a critical role in pathogenesis of bacteria to favor their persistence. Here, we demonstrate that wild type *P. aeruginosa* is a more attractive target for macrophages to adhere to and phagocytize than *lasI-/rhlI-* mutant defective in production of QS signal molecules 3O-C_12_-HSL and C_4_-HSL, and thereby multiply virulence factors (Figure 1). Furthermore, *P. aeruginosa* induced increased expression, distribution and re-organization of AQP9 (Figures 4, 6) paralleled with changes in macrophage size and morphology (Figure 5), that together can facilitate motility, migration and phagocytosis. Thus, we propose novel roles for the QS system and AQP in the interaction between *P. aeruginosa* and human macrophages, which may have an influence on the outcome of infection, inflammation, and development of disease (Figure 8).

*P. aeruginosa* is a highly adaptable Gram-negative bacterium, involved in both acute and chronic infections, typically in patients having compromised epithelial barriers and immune systems or the genetic disorder cystic fibrosis. *P. aeruginosa* is a common cause of lung, skin, ocular, urinary tract, and gastrointestinal tract infections. Experimental models of *P. aeruginosa* in acute pulmonary and burn-wound infections in mice have shown, that strains containing mutations in one or more of the QS genes are less virulent than wild-type bacteria (Tang et al., 1996; Rumbaugh et al., 1999; Pearson et al., 2000). QS signals have been detected in the sputum from cystic fibrosis patients (Singh et al., 2000) typically colonized with *P. aeruginosa, Burkholderia* species and *Staphylococcus aureus*. Clinical *P. aeruginosa* cystic fibrosis isolates often harbor mutations in QS system which gives them advantages to survive in low amino acid pulmonary environment, increased β-lactamase activity and hereby better adaptation during chronic respiratory infections. In contrast, *Burkholderia* QS mutants were far less frequent during chronic infections, which can have a competitive advantage for these species *in vivo* (Cullen and McClean, 2015).

Macrophages may sense and recognize *P. aeruginosa* harboring a functional QS system as a danger signal and thereby perhaps mount more effective phagocytosis to eliminate bacteria (Figure 1). Stimulation of macrophages with AHL was able to only partly elevate phagocytosis of bacteria, suggesting that other factors directly or indirectly controlled by *lasI/rhlI* may play a larger role in this event (Figure 2). Also, this partial effect can be explained by donor-specific variations in macrophage population that display different capacities and functions, including migration and phagocytosis.

Indeed, 3O-C_12_-HSL signal molecules did modulate the level of phagocytosis of yeast particles by macrophages but this was not accompanied by an enhanced respiratory or oxidative burst (Vikström et al., 2005). It is of course also a risk for the host, if the bacteria survive intracellularly. Neutrophils, always appearing at an early stage of infection, can sense 3O-C_12_-HSL and 3O-C_10_-HSL and are strongly stimulated to perform chemotaxis, directional migration (Karlsson et al., 2012) and phagocytosis (Wagner et al., 2007). Interestingly, AHL affect the migration and phagocytosis in a time- and dose- dependent manner. The discrepancies we observe between the different MOI could perhaps in part be explained by the different concentrations of AHL. Later in the presence of mature *P. aeruginosa* biofilms, phagocytes may fail in their orientation and ROS production and migrate on the extracellular polymeric surface of biofilms in a disoriented manner (Bylund et al., 2006; Hansch et al., 2008). The bacteria, on the other hand, might continue to respond aggressively to the presence of leukocytes by up-regulating the production of a number of QS-controlled virulence factors, including rhamnolipids (Jensen et al., 2007), pyocyanine (Allen et al., 2005), and exopolysaccharides (Bylund et al., 2006), which then surround the biofilm and affect incoming immune cells. Moreover, 3O-C_12_-HSL signals have been shown to down-regulate the production of the proinflammatory cytokine TNF-alfa (Telford et al., 1998) and anti-inflammatory cytokine IL-10 in macrophages (Glucksam-Galnoy et al., 2013) and increase IL-8 synthesis in bronchial epithelial cells, leading to massive infiltration of leukocytes (Smith et al., 2001). These bacterial traits illustrate the complex array of QS-dependent communication through which opportunistic bacteria can resist clearance, immune defense, and establish a chronic infection and destructive inflammation.

A first direct evidence for the involvement of AQP in physiological cell migration was made by Loitto et al. (2002), who detected lamellipodial localization of AQP9 during migration. The concentration of a fluorescent cytoplasmic marker was diluted in developing protrusions, indicating a localized influx of water. It was proposed that AQP9 can promote membrane extension by a parallel influxes of water and ions that would then create an increased hydrostatic pressure, forcing the membrane to expand and allowing rapid actin polymerization to fill in a protrusion (Loitto et al., 2002, 2009; Saadoun et al., 2005). Accordingly, we decided to investigate the role of AQP9 during bacterial infection. Indeed, using different imaging technology, we observe that *P. aeruginosa* infection results in increased cellular distribution and re-localization of AQP9 to the leading and trailing regions in human macrophages (Figures 4, 6) and in parallel, distinct changes in macrophage cell size and morphology (Figure 5), that could facilitate motility, migration, and phagocytosis. Remarkably, the bacteria with functional QS system seemed to contribute more to these processes, illustrating the complex array events in host-pathogen communication, potentially contributing to different outcomes of infections and inflammations (Figure 8).

Furthermore, treatment with wild type *P. aeruginosa* and *lasI-/rhlI-* mutant caused significant increase in expression of AQP9 on protein level compared to non-infected control macrophages (Figure 3), supporting our imaging results. On the contrary, mRNA level for AQP9 maintains at the basal expression level (Table S1). The same pattern was detected for AQP4, 5, 6, 7, and 10 while mRNA levels for AQP0, 1, 2, 3, 8, 11, and 12 were slightly affected. It is worth noting, that a strict correlation between gene and protein expressions may not be seen due to the control at many levels, including transcriptional and translational regulation (Vogel and Marcotte, 2012). Among such regulator, small non-coding RNA, or microRNA are induced upon *P. aeruginosa* infection, facilitate survival of bacteria in lung cells (Zhou et al., 2014), target AQP5 and induce lung tissue damage (Zhang et al., 2014).

The classical role of AQP to mediate trans-epithelial fluid transport is well-understood, for example in the urinary concentration mechanisms and gland fluid secretion. AQP are also involved in a variety of other physiological and cellular processes, such as swelling of tissues, neural signal transduction, fat metabolism, cell volume regulation, cell migration, and organelle physiology (Verkman, 2005). A few studies have raised the possibility that AQP are important for immune and epithelial cell function during infection. Thus, deletion of AQP5 in alveolar epithelia led to increased bacterial blood dissemination, reduced mucin production in the lung and aggravated lung injury during *P. aeruginosa* infection, speaking for a protective role of AQP5 (Zhang et al., 2011). Furthermore, during pulmonary adenoviral infection, the levels of AQP1 and 5 were decreased and associated with worsened fluid fluxes (Towne et al., 2000). AQP2 and 3 were proposed to contribute to diarrhea caused by Gram-negative bacteria (Guttman et al., 2007). AQP9 was involved in LPS-induced blood-brain barrier permeability and brain water content (Wang et al., 2009), and identified as a potential marker of chronic inflammation in patients with several different autoimmune diseases (Mesko et al., 2010). In this report, we have discovered and added one more player to infection and inflammatory events, since we noted that *P. aeruginosa* specially one with a working QS system *lasI/rhlI* induces increased AQP9 expression, re-organization and changes in macrophage size.

To conclude, our study demonstrates that the phagocytosis of *P. aeruginosa* by macrophages is more effective when the QS genes *lasI* and *rhlI* being responsible for synthesis of 3O-C_12_-HSL and C_4_-HSL, and associated virulence factors are fully functional. Moreover, the *P. aeruginosa* infections resulted in elevated AQP9 expression and re-localization to the leading and trailing regions in macrophages, increased cell area and length; bacteria with a working QS system *lasI/rhlI* contributed greater to this process (Figure 8). These events may have an influence on the outcome of infection, inflammation, and subsequent development of disease.

## Author contributions

AH has planned and carried out experiments, analyzed the data, evaluated results, and prepared the manuscript. TK evaluated results and prepared the paper. EV is PI of the project, has designed the study, planned, and done experiments, evaluated results, wrote, and finalized the manuscript.

### Conflict of interest statement

The authors declare that the research was conducted in the absence of any commercial or financial relationships that could be construed as a potential conflict of interest.

## Supplementary material

Figure S1 **The number of adherent and ingested wild type *P. aeruginosa* and its *lasI-/rhlI-* mutant by individual human macrophage**. Macrophages were infected with GFP-labeled wild type *P. aeruginosa* or *lasI-/rhlI-* mutant, stained and analyzed by confocal microscopy as shown in Figure 1. **(A)** Quantification of the number of ingested bacteria per macrophage. **(B)** Quantification of the number of bound and ingested bacteria (macrophage-associated bacteria) per individual macrophage. Shown are the mean ± SE of seven independent experiments performed at separate days from different blood donors (color coded and same donors as in Figures 1B,C). The means ± SE are based on 100–200 cells for each condition per experiment

Figure S2 **Super-resolution SIM imaging of AQP9 in macrophages infected with wild type *P. aeruginosa* and its QS mutant**. Macrophages were infected with GFP-labeled (green) wild type *P. aeruginosa*, the *lasI-/rhlI-* mutant or non-infected (Control), for 1 h. Cells were fixed and stained with antibodies against *P. aeruginosa* (blue), rabbit anti-AQP9 and Atto 647N goat anti-rabbit (red) and analyzed by SIM. Bacteria shown in green in these images were completely ingested. Bar 5 μm.

Table S1 **Changes in AQP mRNA levels for macrophages treated with wild type *P. aeruginosa* PAO1 or its *lasI-/rhlI-* mutant at MOI 1, 10 and 100 for 1h compared to levels for the control untreated cells**.

## Abbreviations

3O-C_12_-HSL: *N*-3-oxododecanoyl-homoserine lactone
3O-C_10_-HSL: *N*-dodecanoyl-homoserine lactone
C_4_-HSL: *N*-butyryl-*L*-homoserine lactone
AHL: *N*-acylhomoserine lactone
AQP: aquaporin(s)
DMSO: dimethylsulfoxide
F-actin: filamentous actin
G-actin: globular actin
IL: interleukin
KRG: Krebs Ringer Glucose buffer
KRG+Ca^2+^: calcium-containing Krebs-Ringer Glucose buffer
LPS: lipopolysaccharide
LSCM: laser scanning confocal microscopy
MOI: multiplicities of infection
NA: numerical aperture
RT: room temperature
SIM: structured illumination super-resolution microscopy
STED: stimulated emission depletion super-resolution microscopy/nanoscopy
QS: quorum sensing
TNF: tumor necrosis factor.
