# Supplementary figures and images for: Pseudomonas aeruginosa lasI/rhlI quorum sensing genes promote phagocytosis and aquaporin 9 redistribution to the leading and trailing regions in macrophages
Source: Front Microbiol. 2015 Sep 3;6:915. doi: 10.3389/fmicb.2015.00915 (PMC4558532; doi:10.3389/fmicb.2015.00915)

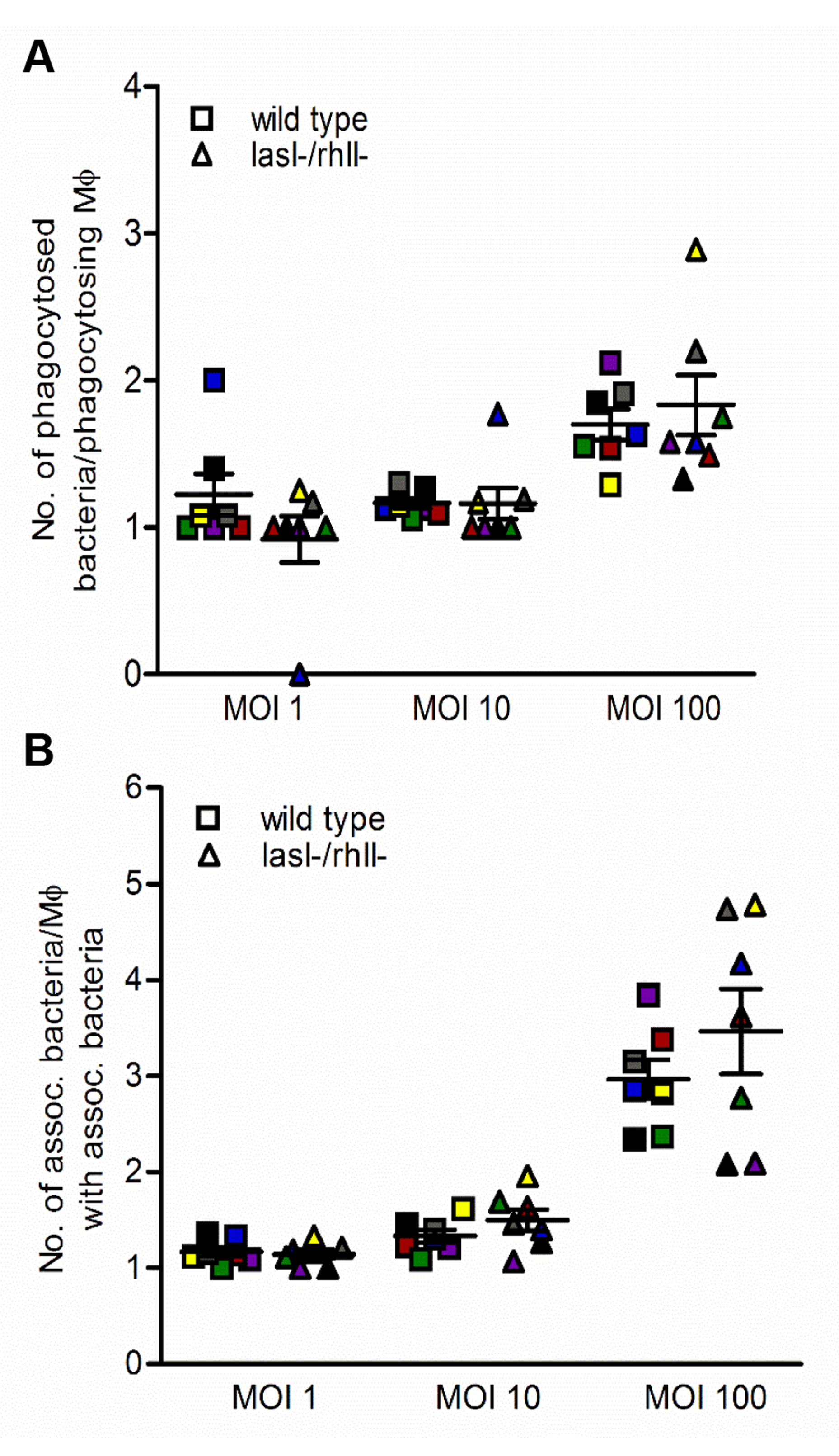

Supplement: Figure S1 — The number of adherent and ingested wild type P. aeruginosa and its lasI-/rhlI- mutant by individual human macrophage. Macrophages were infected with GFP-labeled wild type P. aeruginosa or lasI-/rhlI- mutant, stained and analyzed by confocal microscopy as shown in Figure 1. (A) Quantification of the number of ingested bacteria per macrophage. (B) Quantification of the number of bound and ingested bacteria (macrophage-associated bacteria) per individual macrophage. Shown are the mean ± SE of seven independent experiments performed at separate days from different blood donors (color coded and same donors as in Figures 1B,C). The means ± SE are based on 100–200 cells for each condition per experiment [file Image1.TIF]

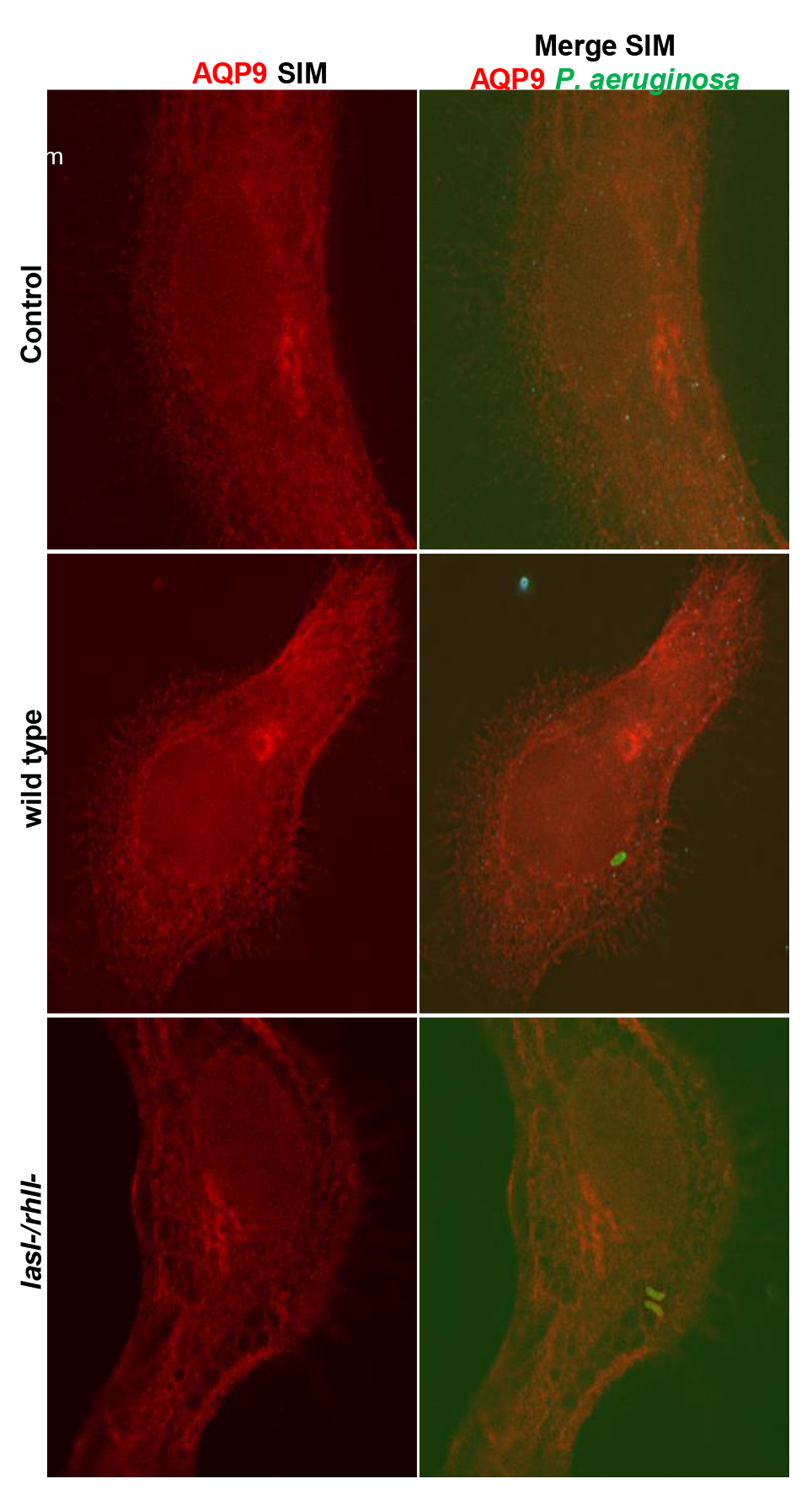

Supplement: Figure S2 — Super-resolution SIM imaging of AQP9 in macrophages infected with wild type P. aeruginosa and its QS mutant. Macrophages were infected with GFP-labeled (green) wild type P. aeruginosa, the lasI-/rhlI- mutant or non-infected (Control), for 1 h. Cells were fixed and stained with antibodies against P. aeruginosa (blue), rabbit anti-AQP9 and Atto 647N goat anti-rabbit (red) and analyzed by SIM. Bacteria shown in green in these images were completely ingested. Bar 5 μm. [file Image2.TIF]
