# Supplementary material for: Pseudomonas aeruginosa lasI/rhlI quorum sensing genes promote phagocytosis and aquaporin 9 redistribution to the leading and trailing regions in macrophages
Source: Front Microbiol. 2015 Sep 3;6:915. doi: 10.3389/fmicb.2015.00915 (PMC4558532; doi:10.3389/fmicb.2015.00915)
Supplement: Table S1 — Changes in AQP mRNA levels for macrophages treated with wild type P. aeruginosa PAO1 or its lasI-/rhlI- mutant at MOI 1, 10 and 100 for 1h compared to levels for the control untreated cells. [file Table1.DOCX]

**Table S1.** Changes in AQP mRNA levels for macrophages treated with wild type *P. aeruginosa* PAO1 or its *lasI-/rhlI-* mutant at MOI 1, 10 and 100 for 1h compared to levels for the control untreated cells.

| Protein (Uniprot accession number) | Gene (Uniprot accession number) | Protein description and its subcellular location | Mediates passage of | Absolute fold changes | | | | | |
| --- | --- | --- | --- | --- | --- | --- | --- | --- | --- |
|  |  |  |  | WT  /control  MOI 1 | WT  /control  MOI 10 | WT  /control  MOI 100 | lasI-/rhlI-/control  MOI 1 | lasI-/rhlI-/control  MOI 10 | lasI-/rhlI-/control  MOI 100 |
| [P30301](http://www.uniprot.org/uniprot/P30301) | AQP0 | AQP0  membrane | Water | -1.5 | -6.9 | -6.9 | -6.9 | -5.8 | -6.9 |
| [P29972](http://www.uniprot.org/uniprot/P29972) | AQP1 | AQP1  membrane | Water | 1.0 | 1.4 | 2.2 | 1.0 | 1.0 | 1.0 |
| [P41181](http://www.uniprot.org/uniprot/P41181) | AQP2 | AQP2  membrane, cytoplasmic vesicle membrane, Golgi, ER | Water | 5.2 | 2.1 | 1.2 | 6.9 | 1.0 | 2.2 |
| [Q92482](http://www.uniprot.org/uniprot/Q92482) | AQP3 | AQP3  membrane | Water  Glycerol  Urea | -1.9 | -2.8 | -2.8 | -1.1 | 1.7 | 2.5 |
| [P55087](http://www.uniprot.org/uniprot/P55087) | AQP4 | AQP4  membrane | Water | 1.0 | 1.0 | 1.0 | 1.0 | 1.0 | 1.0 |
| [P55064](http://www.uniprot.org/uniprot/P55064) | AQP5 | AQP5  membrane | Water | 1.0 | 1.0 | 1.0 | 1.0 | 1.0 | 1.0 |
| [Q13520](http://www.uniprot.org/uniprot/Q13520) | AQP6 | AQP6  cytoplasmic vesicle membrane | Water  Anion | 1.0 | 1.0 | 1.0 | 1.0 | 1.0 | 1.0 |
| [O14520](http://www.uniprot.org/uniprot/O14520) | AQP7 | AQP7  membrane | Water  Glycerol  Urea | 1.0 | 1.1 | 1.0 | 1.0 | 1.0 | 1.0 |
| [O94778](http://www.uniprot.org/uniprot/O94778) | AQP8 | AQP8  membrane | Water | 1.0 | 1.0 | 1.6 | 1.0 | 7.2 | 2.4 |
| [O43315](http://www.uniprot.org/uniprot/O43315) | AQP9 | AQP9  membrane | Water  Glycerol  Urea  Non-charged solutes | -1.1 | -1.2 | -1.1 | -1.2 | -1.2 | -1.1 |
| [Q96PS8](http://www.uniprot.org/uniprot/Q96PS8) | AQP10 | AQP10  membrane | Water  Glycerol  Urea | 1.0 | 1.0 | 1.0 | 1.0 | 1.0 | 1.0 |
| [Q8NBQ7](http://www.uniprot.org/uniprot/Q8NBQ7) | AQP11 | AQP11  Membrane  ER | Water  Glycerol  Urea  Small neutral solutes | 1.4 | 1.7 | 1.0 | 2.1 | -3.0 | -1.1 |
| [Q8IXF9](http://www.uniprot.org/uniprot/Q8IXF9) | AQP12A | AQP12A  membrane | Water  Glycerol  Urea  Small neutral solutes | -2.9 | -2.9 | -2.9 | -2.0 | -2.9 | -2.9 |
| [A6NM10](http://www.uniprot.org/uniprot/A6NM10) | AQP12B | AQP12B  membrane | Water  Glycerol  Urea  Small neutral solutes | 1.1 | -1.3 | -1.3 | -1.2 | 1.2 | -1.1 |
| P04406 | GAPDH | GAPDH | Glyceraldehyde-3-phosphate dehydrogenase | -1.1 | -1.1 | -1.1 | -1.1 | -1.1 | -1.1 |
